# Transient Developmental Expression of IgY and Secretory Component like Protein in the Gut of the Axolotl (*Ambystoma mexicanum* )

**DOI:** 10.1155/1992/21679

**Published:** 1992

**Authors:** J. S. Fellah, S. Iscaki, J. P. Vaerman, J. Charlemagne

**Affiliations:** 1 Laboratoire d’Immunologie Comparée Université Pierre et Marie Curie and CNRS 9 quai Saint-Bernard Paris 75005 France; 2 :Unité d’Immunologie Microbienne Institut Pasteur 25-28 rue du Docteur Roux Paris 75724 France; 3 International Institute of Cellular and Molecular Pathology Unit of Experimental Medicine Université Catholique de Louvain 75 avenue Hippocrate Brussels B-1200 Belgium

**Keywords:** IgY, Secretory immunoglobulins, tissue distribution, amphibian development, axolotl, ontogeny

## Abstract

We previously reported that a primitive vertebrate, the Mexican axolotl (Amphibian, Urodela) synthesizes two classes of immunoglobulins. IgM are present in serum early in the development, and represent the bulk of specific antibody synthesis after an antigenic challenge. IgY occur in the serum later during the development, and are relatively insensitive to immunization. We demonstrate in the present work, using immunofluorescence with specific Mabs, that IgY are expressed in the gut epithelium, as secretory molecules. Secretory IgY are well expressed in the stomach and intestinal mucosae of young animals from 1 month after hatching to the seventh month. Thereafter, IgY progressively disappear from the gut and become readily detectable in the serum of 9-month-old preadult immunologically mature animals. Axolotl IgY are closely associated in the gut to secretory component-like (SC) molecules that are well-recognized by antisera to the SC of different mammalian species. This is the first description, in a primitive tetrapode, of an immunoglobulin class that could be the physiological counterpart of mammalian IgA.


*Developmental Immunology*, 1992, Vol. 2, pp. 181–190
Reprints available directly from the publisher
Photocopying permitted by license only

© 1992 Harwood Academic Publishers GmbH
Printed in the United Kingdom

### Transient Developmental Expression of IgY and Secretory Component like Protein in the Gut of the Axolotl (*Ambystoma mexicanum*)

J. S. FELLAH,*† S. ISCAKI,‡ J. P. VAERMAN,§ and J. CHARLEMAGNE†

†*Laboratoire d'Immunologie Comparée, Université Pierre et Marie Curie and CNRS, 9 quai Saint-Bernard, 75005 Paris, France*
‡*Unité d'Immunologie Microbienne, Institut Pasteur, 25-28 rue du Docteur Roux, 75724 Paris*
§*International Institute of Cellular and Molecular Pathology, Unit of Experimental Medicine, Université Catholique de Louvain, 75 avenue Hippocrate, B-1200 Brussels, Belgium*

We previously reported that a primitive vertebrate, the Mexican axolotl (Amphibian, Urodela) synthesizes two classes of immunoglobulins. IgM are present in serum early in the development, and represent the bulk of specific antibody synthesis after an antigenic challenge. IgY occur in the serum later during the development, and are relatively insensitive to immunization.

We demonstrate in the present work, using immunofluorescence with specific Mabs, that IgY are expressed in the gut epithelium, as secretory molecules. Secretory IgY are well expressed in the stomach and intestinal mucosae of young animals from 1 month after hatching to the seventh month. Thereafter, IgY progressively disappear from the gut and become readily detectable in the serum of 9-month-old preadult immunologically mature animals.

Axolotl IgY are closely associated in the gut to secretory component-like (SC) molecules that are well-recognized by antisera to the SC of different mammalian species.

This is the first description, in a primitive tetrapode, of an immunoglobulin class that could be the physiological counterpart of mammalian IgA.

KEYWORDS: IgY, Secretory immunoglobulins, tissue distribution, amphibian development, axolotl, ontogeny.

### INTRODUCTION

Protection of the mammalian gastrointestinal mucosae against pathogenic agents largely depends on nonimmune factors such as pH (gastric acidicity), enzymatic secretions (proteases, peroxidase, lysozyme), mobility of the glandular epithelium, and mechanical properties of the epithelial mucus (reviewed in Pabst, 1987). The barrier function of the gut also consists in lymphoid cells, either organized in lymphoid structures such as Peyer's patches, or dispersed in the epithelium (intraepithelial lymphocytes, IEL). The majority of IEL lymphocytes express the T-suppressor/cytotoxic phenotype, whereas T cells in the *lamina propria* are mainly of the T-helper subset (Cerf-Bensussan et al., 1983; Hirata et al., 1986). Most gut-associated B lymphocytes secrete Ig of the IgA class. Secreted IgA molecules consist of IgA-dimers covalently linked to a J chain and associated to a glycoprotein, the secretory component (SC) (Tomasi et al., 1965; Lemaître-Coelho et al., 1977a; Kühn and Kraehenbühl, 1979). SC is the extracellular moiety of a transmembrane receptor (the Poly-Ig receptor) that allows cytoplasmic translocation of IgA (and IgM) from the basolateral to the apical side of enterocytes and the secretion of the translocated molecules in the digestive lumen (Brandtzaeg, 1973, 1981; Nagura et al., 1979). The Poly-Ig receptor is encoded by a gene belonging to the Ig gene superfamily (Mostov et al., 1984).

Secretory immunoglobulins were also observed in several nonmammalian species, including birds (Bienenstock et al., 1973; Lebacq-Verheyden et al., 1972), reptiles (Hädhge et al., 1980), amphibians (Hsu et al., 1985), and fish (reviewed in Hart et al., 1988).

We recently described in the axolotl, a neotenic

*Corresponding author.

urodele amphibian, a previously uncharacterized LMW Ig class that we related to IgY (Fellah and Charlemagne, 1988). These 11.9 S IgY molecules are recognized by a monoclonal antibody (Mab 33.39.2) specific for the H$\psi$ chains. Two independent populations of B lymphocytes synthesizing IgM (B$\mu$) or IgY (B$\psi$) are present in the spleen of younger than 2-month-old axolotls, but IgY molecules are not found in the serum before the ninth month. However, high molecular weight (HMW) IgM are present in the serum of 2-month-old animals (Fellah et al., 1989).

The present work describes the transitory expression, during the first 7 months of development, of IgY in the axolotl gastrointestinal tract, and their association with molecules antigenically related to mammalian SC.

### RESULTS

### Immunochemical Localization of IgY in the Axolotl Gut

Ig distribution was investigated in the axolotl gut using indirect immunofluorescence staining on paraffin sections. Strongly fluorescent structures appeared using Mab 33.39.2 (anti-H$\psi$), with some variations during larval development. In stomach sections of young axolotls (1 month after fertilization), a bright staining occurred in the cytoplasm of large cells located in the *lamina propria* near the basal side of enterocytes (Fig. 1, panels 1 and 2). These cells correspond to the stomach serous or fundic gland structures. Cells of the digestive epithelium were not labeled at this stage.

In 1.5-month-old axolotl, the basal side of epithelium columnar cells begins to react with Mab 33.39.2. The stomach glands are then completely differentiated and remain strongly labeled (Fig. 1, panels 3 and 4).

In elder animals (3 to 7 months), labeling was also observed in the lumen of the secretory glands. The basal area and the apical cytoplasm of columnar cells fluoresced brightly; both crypt and surface epithelial cells were stained by 33.39.2 (Fig. 1, panels 5 and 6). No reactivities were noticed in *muscularis mucosae* and submucosal structures. From the seventh month on, labeling weakened and completely disappeared from the stomacal mucosae at 9 months (Fig. 1, panels 7 and 8).

In intestinal mucosae, IgY were detected at the three levels: duodenum, jejunum, and ileum. The apical cytoplasm, the brush borders, and the basal side of columnar cells fluoresced brightly with Mab 33.39.2 (Fig. 2, panels 1 to 4). Goblet cells were never stained. Like in stomach, the reactivity against H$\psi$ chains in other segments of the digestive tract was completely abolished at 9 months (Fig. 2, panels 5 and 6).

As a specificity control, some sections of axolotl gut were incubated with Mab 33.39.2 in the presence of purified axolotl Igs (50–100 $\mu$g/mL). Under these conditions, no staining was observed.

Mab 33.101.2, which recognizes a light-chain determinant of axolotl Igs, and a rabbit antiserum (L-126) specific for noncarbohydrate determinants of the two axolotl Ig isotypes (Fellah and Charlemagne, 1988) were also used for labeling of the axolotl gastrointestinal tract. Although less intense, the fluorescence patterns appeared similar to labels using Mab 33.39.2. This is a clear indication that the revealed activities were due to recognition of immunoglobulin molecules.

No labeling was detected in the axolotl gut at any developmental stage with Mab 33.45.1, specific for the IgM $\mu$ chain (Fig. 3).

### SC-like Expression in the Axolotl Gut

In order to examine the presence of SC-like molecules associated with the axolotl gut, three polyclonal antibodies to human SC (230, 319, and

FIGURE 1. Immunofluorescence detection of secretory IgY in the axolotl stomacal mucosae with Mab 33.39.2. Panels 1, 3, 5, and 7: sections seen in phase contrast. Panels 2, 4, 6, and 8: revelation with Mab 33.39.2. Magnification×600. In 3-week-old axolotl stomach (panels 1 and 2), IgY are concentrated in the cytoplasm of large glandular cells (arrows) forming acini, located in the *lamina propria*. Epithelial cells (ec) are not labeled. Two weeks later (panels 3 and 4), the basal side of the epithelium begins to react with Mab 33.39.2, however, apical surfaces are not stained. In 5-month-old axolotl stomach (panels 5 and 6), the apical and basal surfaces of epithelial cells and the glandular lumens appear strongly fluorescent. After the eighth month (panels 7 and 8), the anti-IgY labeling completely disappears from the stomacal mucosae. L: lumen.

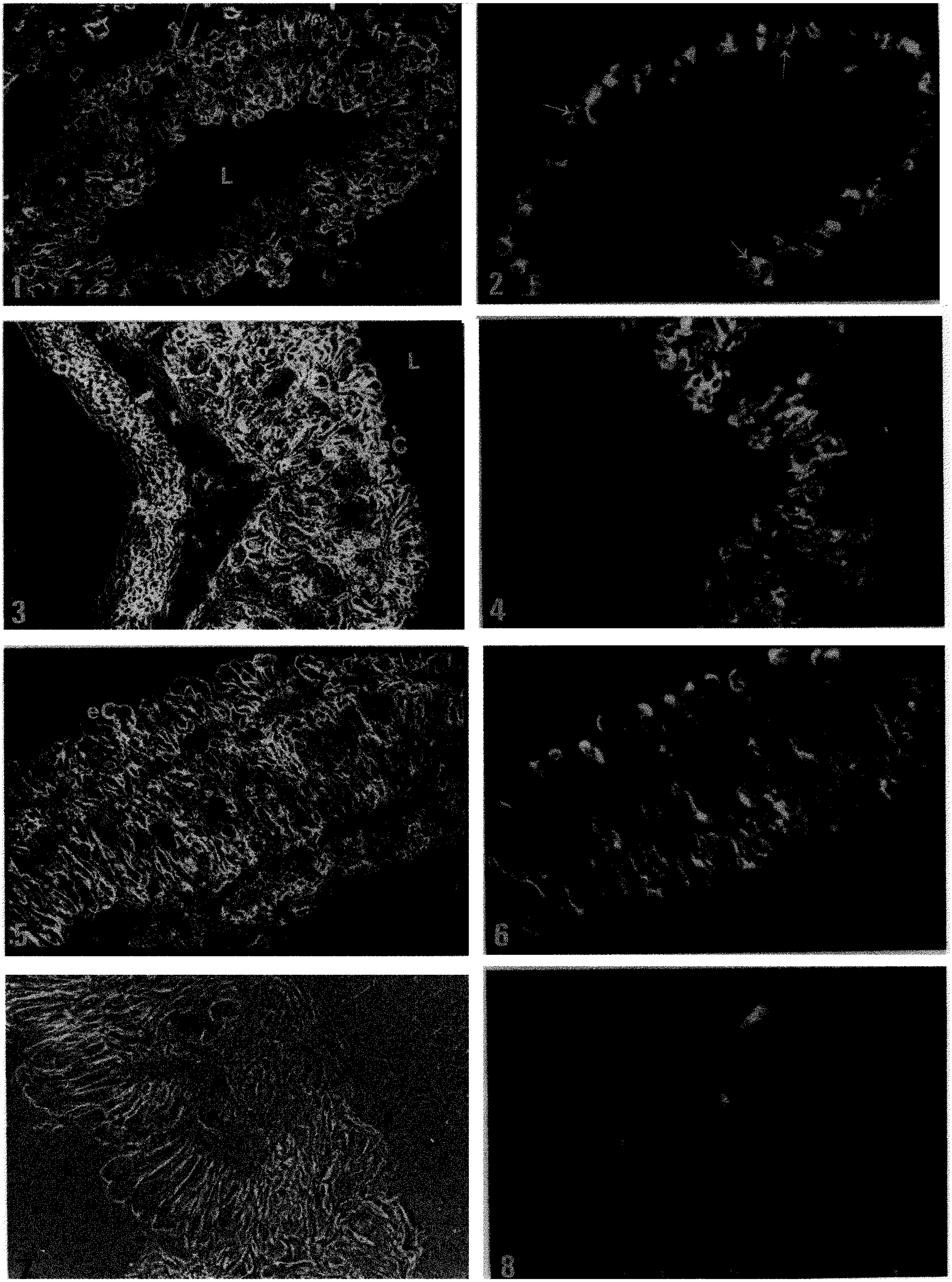

FIGURE 1

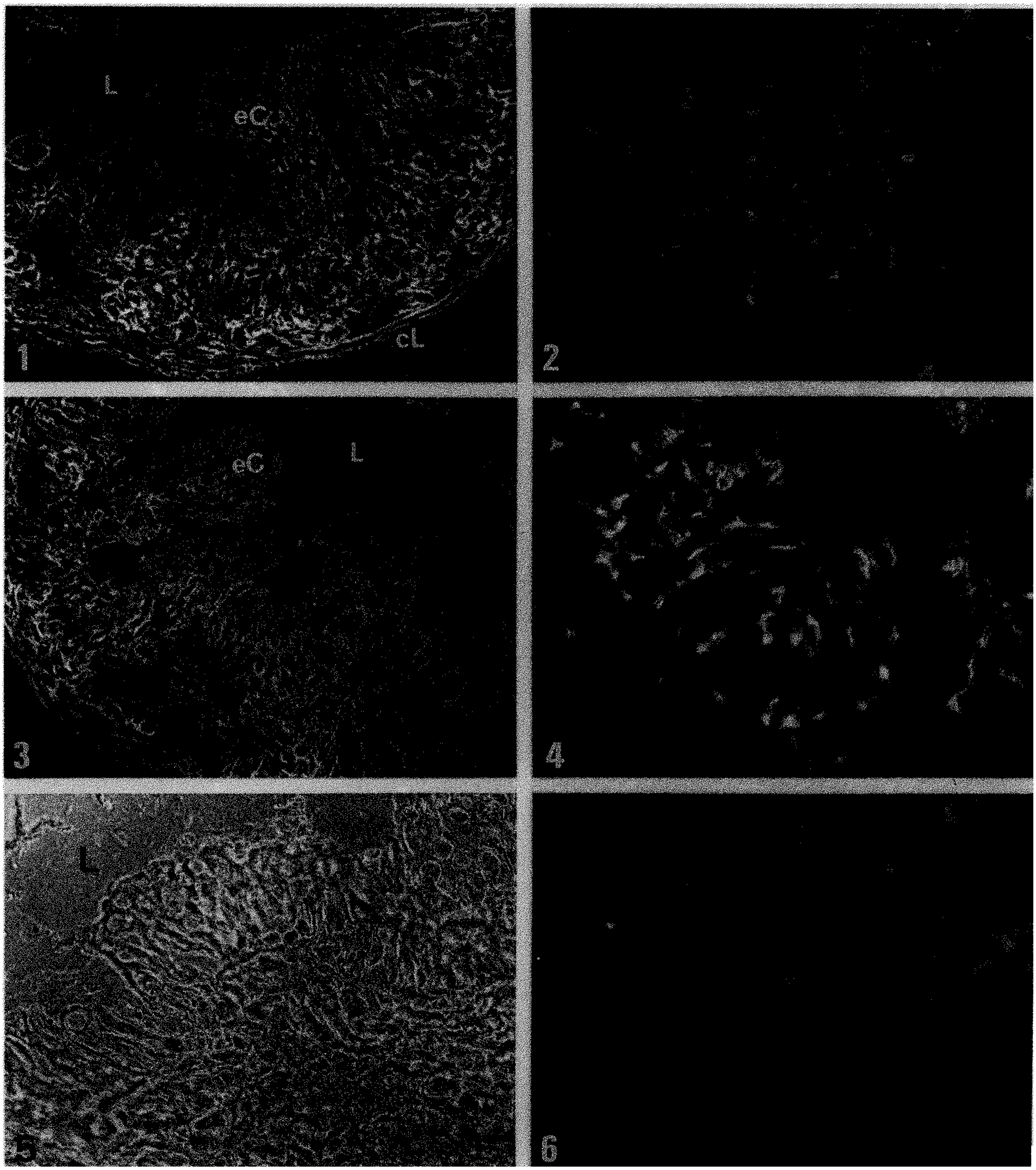

FIGURE 2. Immunofluoresence detection of secretory IgY in the axolotl intestinal mucosae with Mab 33.39.2. Panels 1, 3, and 5: sections seen in phase contrast. Panels 2, 4, and 6: revelation with Mab 33.39.2. Magnification×600. In 5-week-old axolotl (panels 1 and 2), labeling is essentially located in the cytoplasm of epithelial cells. Apical poles and membranes are not labeled at this stage. At 5 months (panels 3 and 4), both cytoplasms and pole surfaces of epithelial cells appear clearly labeled. The muscular coat and the conjunctive layer are not labeled. At 9 months (panels 5 and 6), no staining is detected in the intestinal mucosae. cL: conjunctive layer; eC: epithelial cells; L: lumen.

TRY) were tested by indirect immunofluorescence in paraffine sections of stomach and intestinal tissues.

A strong fluorescence labeling was obtained with both anti-SC 230 and TRY antisera. In stomach of 5-month-old axolotls, labeling was concentrated essentially in glandular cells and in the apical part of epithelial cells (Fig. 4, panels 1 and 2). In intestinal sections, the fluorescence pattern was distributed on glandular structures and at the basal, apical, and cytoplasmic levels of enterocytes.

By using double immunofluorescence, tissue sections were stained with anti-SC antibodies and with Mab 33.39.2. A complete superposal of the histological structures labeled by these two antibodies was observed, suggesting a physical association of the recognized molecules (Fig. 4, panels 5 and 6).

The temporal expression of SC-like molecules in the axolotl gut during ontogenesis appeared slightly different from the expression of secretory IgY. In 2-month-old animals, SC-like expression was observed in both glandular cells and enterocytes. After the seventh month, although the fluorescence intensity progressively decreased, staining was never completely abolished. Thus, in adult axolotl (12–16-month-old), a residual faint labeling was always observed at the apical side (brush borders) of columnar cells (Fig. 4, panels 3 and 4).

The anti-SC antiserum 319 (which mainly recognizes the accessible determinants of SC, see Materials and Methods) has no reactivity for the axolotl gastrointestinal tract.

To confirm the presence of an SC-like molecule in the axolotl gut, several polyclonal antibodies directed against bile or milk mammalian SC were

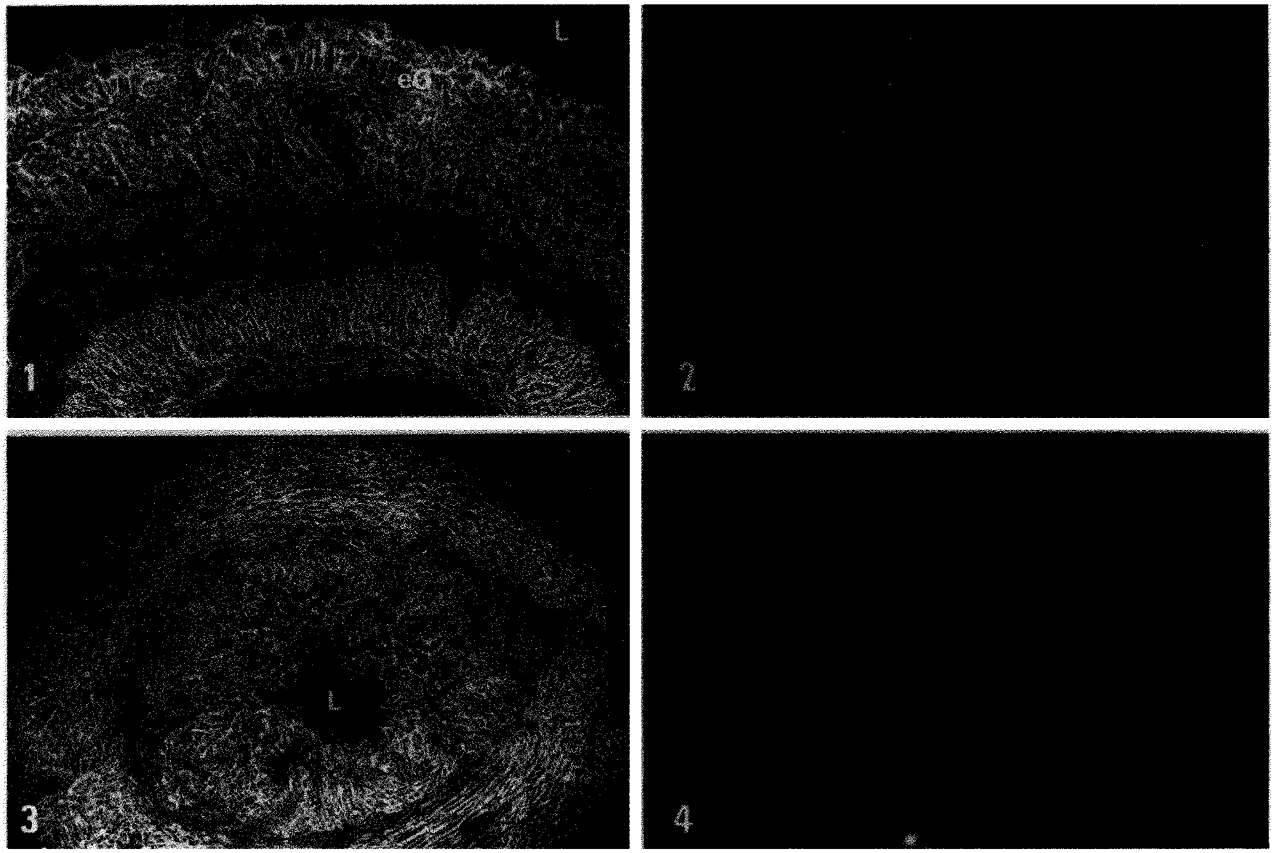

FIGURE 3. Immunofluorescence detection of IgM in axolotl stomach (panels 1 and 2) are intestine mucosae (panels 3 and 4). Panels 1 and 3: sections seen in phase contrast. Panels 2 and 4: revelation with Mab 33.34.1. Magnification×600. This experiment indicates that IgM are not expressed in the axolotl digestive tract, although in the same experimental conditions, intraepithelial IgY are easily detected. L: lumen.

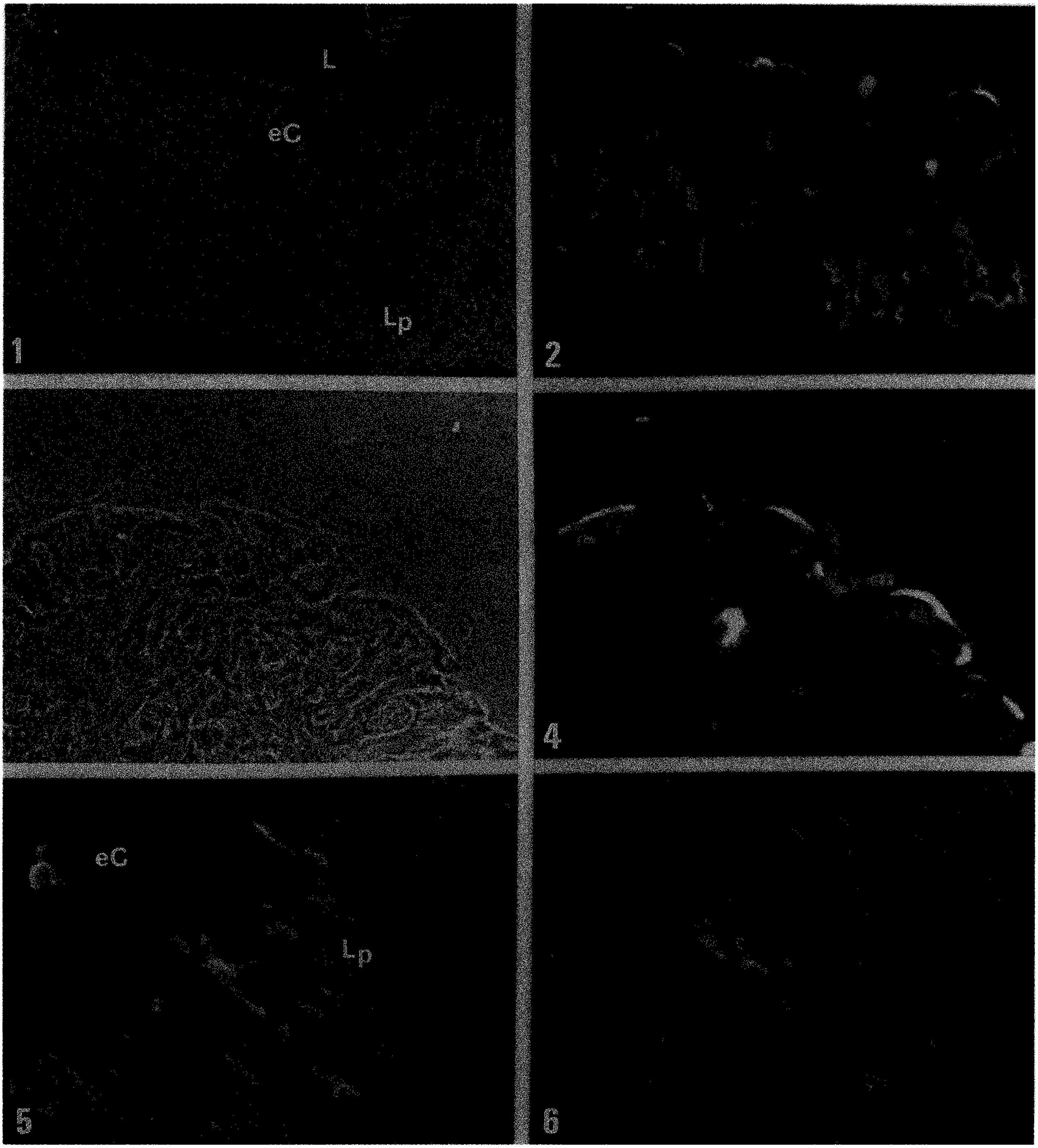

FIGURE 4. Immunofluoresence staining of stomacal mucosae with the antihuman SC sheep 230 antiserum. Panels 1 and 3: sections seen in phase contrast. Panels 2 and 4: revelation with antiserum 230. Panels 5 and 6: double immunofluoresence using Mab 33.39.2 (5) and antiserum 230 (6). Magnification×600. In 5-month-old stomach (panels 1 and 2), a bright labeling of the membrane and cytoplasm of glandular cells is observed. The apical and basal sides of epithelial cells (eC) are also strongly revealed. At 12 months (panels 3 and 4), only the apical poles of epithelial cells are slightly labeled. The double immunofluorescence experiment using Mab 33.39.2 (panel 5) and antiserum 230 (panel 6) on the same section of a 5-month-old axolotl stomach reveals a perfect superposal pattern of both labels. L: lumen; Lp: *lamina propria.*

TABLE 1
Origins, Specificities, and Anti-axolotl Reactivities (as Estimated by Immunofluoresence Intensity) of the Various Anti-SC Antibodies Used

| Antibody to | Code number | Made in | Reactivity with axolotl gut |
|---|---|---|---|
| Human milk SC | 230[a] | sheep | ++++ |
| Human milk SC | 319 | sheep | – |
| Human milk SC | TRY | rabbit | +++ |
| Human milk SC | L 519 | rabbit | +/– |
| Human milk SC | Ch 606 | goat | + |
| Rat bile SC | L 858 | rabbit | – |
| Rat bile SC | Ch 657 | goat | +++ |
| Mouse milk SC | L 863 | rabbit | – |
| Guinea pig milk SC | L 709 | rabbit | – |
| Dog milk SC | Ch 607 | goat | ++ |

[a]References for antisera 230, 319, and TRY are given in Materials and Methods. References for the other antisera are Delacroix and Vaerman, 1981 (Ch 606); Vaerman et al., 1975a (L 858); Acosta-Altamirano et al., 1980 (Ch 657); Lemaître-Coelho et al., 1977b (L 863); Vaerman et al., 1975b (L 709); Delacroix et al., 1983 (Ch 607). Antiserum L 519 (from J.P.V) was not previously described.

tested, and the results are summarized in Table 1. The staining patterns observed with goat antisera 657 and 606, and with rabbit antiserum L 519, were similar to those given by antiserum 230, the fluorescence appearing, however, to be much weaker.

### DISCUSSION

Our results demonstrate the presence of IgY in the axolotl gastrointestinal tract, using a well-characterized Mab (33.39.2) specific for the H$\psi$ component of these molecules. These observations are confirmed by the recognition of the same structures by Mab 33.101.2, which is specific for a light-chain epitope of axolotl IgY and IgM, and by a rabbit antiserum (L-126) recognizing all the polypeptide components of both Ig classes. On the other hand, Mab 33.45.1, specific for the H$\mu$ component of axolotl IgM, never reacted with gut mucosal structures. These Mabs and the antiserum L-126 were widely used in our previous works for the detection of different lymphoid subpopulations using immunohistological techniques (Fellah, 1989; Fellah et al., 1989). Their specificities were always clear and selective, and they never stained, except for B lymphocytes, any epidermic (including mucous glands), mesodermic, or nervous structure, even in backgrounds.

Staining of 1-month-old axolotl gut structures was bright and precisely located in glandular structures, forming acini in stomach and intestine submucosae. The histological observation of these glands in axolotl larvae indicates that they are in continuity with the digestive epithelium. One month later, labels were also found associated to basal and apical parts of absorptive epithelial cells. By comparison, immunohistochemical studies of IgA distribution in the mammalian gut mucosae show a similar distribution: the epithelium labeling for IgA was seen to be related to the basolateral surface of columnar cells and found in the apical cytoplasm (reviewed in Brandtzaeg, 1974).

In 2-month-old axolotls, although differentiated B$\mu$ and B$\psi$ lymphocytes were present in the spleen, only IgM molecules were secreted in serum. Histological studies of axolotl intestine and stomach sections revealed the presence of rare lymphoid cells in *lamina propria* and epithelium, but cells of the plasmocytic series were not seen (Fellah et al., 1989). This suggests that in the axolotl, secretory IgY are not locally produced by gut-associated B cells.

Five different antisera against human or rat SC were tested on axolotl gut sections and showed staining patterns similar to that obtained using Mab 33.39.2. These results were confirmed by double fluorescence experiments giving patterns similar to those described in the mammalian intestinal mucosae when using SC and IgA antibodies (Brandtzaeg, 1974). After the seventh month, the expression of SC in axolotl decreased in the same way as that of intraepithelial IgY.

In this study, significant immunofluorescence reactions using polyclonal anti-SC antisera were never observed at the level of mesodermic structures like blood vessels, connective tissues, or muscular structures. Skin epithelial structures and associated glands were not stained. These antisera never reacted against axolotl Igs in ELISA or Western blotting (data not shown) and were controlled to be unreactive for free mammalian IgA (Geneste et al., 1986). Antiserum 230 (anti-human milk SC) appeared highly specific at low dilutions (1/2000) and never reacted with axolotl leukocytes.

Preliminary immunoblotting experiments indicate that antiserum 230 reveals a 78 kD polypeptide in young axolotl stomach and intestine extracts, in good correlation with the estimated molecular weight of mammalian SC (Kühn and Kraehenbühl, 1981).

In mammals, only the dimeric forms of IgA molecules locally secreted by gut plasma cells, or coming from the serum, can bind to the epithelial Poly-Ig receptors, whereas IgA monomers cannot. In the rat, mouse, rabbit, and chicken, a dimeric form of serum IgA can be transported from blood to bile (reviewed in Underdown and Schiff, 1986). The sedimentation constant of serum axolotl IgY (11,9 S) suggests a dimeric form and thus the capacity to be directly captured by the epithelial secretory system. However, it is still not known if the axolotl IgM and IgY do or do not possess a J chain, a prerequisite for strong binding of mammalian Igs to SC.

The transitory expression of high levels of Poly-Ig receptor-like molecules, associated to the basal membrane of the digestive epithelium in young axolotls, could be an explanation for the inverse correlation between the IgY levels in gut and serum. One to three weeks after hatching, a small number of B$\psi$ and B$\mu$ appear in the spleen and IgM, but no IgY can be detected in serum. Thus, in young immunologically immature animals, the total secreted igY input brought by lymphatic and blood circulations may be captured by the gut secretory system and serve to protect the intestinal barrier in the absence of more specific immune responses. Since the seventh month, a lower expression of Poly-Ig receptors may allow the progressive accumulation of IgY molecules in serum.

### MATERIALS AND METHODS

### Animals

Axolotls (*Ambystoma mexicanum*) of the Ax6 black strain were bred in our laboratory colony in 14 °–16 °C tap water and fed twice a week with commercial fish pellets. About 100 animals from 1-month-old to adult (17-month-old) were used in this study.

### Antibodies

Obtention and characterization of Mabs specific for axolotl Ig chains have already been described (Chardin et al., 1987; Fellah and Charlemagne, 1988). Mabs 33.45.1 and 33.39.2 are specific for the $\mu$ and $\psi$ heavy-chain isotypes, respectively. Mab 33.101.2 recognizes an Ig light-chain determinant shared by both Ig classes.

Polyclonal SC-specific antisera 230 and 319 were obtained from sheep hyperimmunized with purified human colostral SC and their fine specificities have been previously described (Iscaki et al., 1978; Geneste et al., 1986). Briefly, antiserum 230 recognizes four SC antigenic determinants [A1, A2 (accessible when SC is combined with IgA), I (hidden), and R (partially hidden),] whereas antiserum 319 is mainly directed against the accessible determinants of SC.

An antiserum (rabbit TRY) specific for the A2 and weakly for the A1 determinants of SC was also used (Geneste et al., 1986).

A set of polyclonal antibodies directed against milk and/or bile SC of different mammalian species was also used and its origins and specificities are listed in Table 1.

To remove potential natural antibody activities against axolotl cell-surface determinants, all the anti-SC antisera were absorbed twice, for 30 min at 4 °C with packed axolotl erythrocytes (v/v), before use.

### Immunohistology

Organs were washed in amphibian phosphate-buffered saline (A-PBS, Du Pasquier et al., 1972) and fixed for 24 hr in paraformaldehyde-lysine sodium periodate (PLP, Gendelman et al., 1983). Pieces were then washed for 24 hr in A-PBS, dehydrated in isopropanol for 15 hr and cleared for 15 min in chloroform. The procedure was performed at 4 °C. Tissues were then embedded in paraffin. Sections (7 $\mu$m) were floated onto slides previously coated with 0.01% poly-L-lysine (Sigma) and stored for 24 hr at room temperature before use.

For immunofluorescence staining, the deparaffined sections were washed twice in A-PBS, saturated for 30 min with 3% defatted dry milk in A-PBS and incubated with the first antibody. After three washes in A-PBS, sections were incubated with the FITC-labeled second antibody previously absorbed with axolotl leucocytes.

For double immunofluorescence labeling, after the blocking steps, sections were incubated successively with the first antibody (Mab anti-axolotl Ig), biotinylated rabbit anti-mouse IgG (1/200 in A-PBS, Amersham), Texas red conjugated avidine (1/50 in A-PBS, Amersham), the second antibody (sheep anti-human SC), and, finally, FITC-conjugated goat anti-sheep antibodies (1/200 in A-PBS, Sigma). Each incubation step was followed by three washes in A-PBS. Sections were mounted in Mowiol (Hoechst) and observed by epifluorescence UV microscopy.

### ACKNOWLEDGMENTS

This work was supported in part (J.P.V.) by grant number 3.4549.87 from the "Fonds de la Recherche Scientifique Médicale," Brussels, Belgium.

We thank Geneviève Aubet and Jean Desrosiers for excellent technical assistance and Brigitte Cuvelier for the preparation of the manuscript.

*(Received June 11, 1991)*

*(Accepted September 24, 1991)*

### REFERENCES

Acosta-Altamirano G, Barranco-Acosta C., van Roost E., and Vaerman J.P. (1980). Isolation and characterization of secretory IgA (sIgA) and free secretory component (FSC) from rat bile. Molec. Immunol. **17:** 1525–1534.

Bienenstock J., Perey D.Y.E., Gauldie J., and Underdown B.J. (1973). Chicken IgA: Physicochemical and immunochemical characterization. J. Immunol. **110:** 524–533.

Brandtzaeg P. (1973). Structure, synthesis and external transfer of mucosal immunoglobulins. Ann. Immunol. (Inst. Pasteur) **124C:** 417–448.

Brandtzaeg P. (1974). Mucosal and glandular distribution of immunoglobulin components. Differential localization of free and bound SC in secretory epithelial cells. J. Immunol. **112:** 1553–1559.

Brandtzaeg P. (1981). Transport models for secretory IgA and secretory IgM. Clin. Exp. Immunol. **44:** 221–232.

Brandtzaeg P. (1987). Translocation of immunoglobulins across human epithelia: Review of the development of a transport model. Acta Histochemica **8:** 9–32.

Cerf-Bensussan N., Schneeberger E.E., and Bhan A.K. (1983). Immunohistologic and immunoelectron microscopic characterization of the mucosal lymphocytes in human small intestine by the use of monoclonal antibodies. J. Immunol. **130:** 2615–2622.

Chardin H., Vilain C., and Charlemagne J. (1987). Characterization of axolotl heavy and light immunoglobulin chains by monoclonal antibodies. Hybridoma **6:** 627–635.

Delcroix D.L., Furtado-Barreira G., de Hemptinne B., Goudswaard J., Rahier J., Dive C., and Vaerman J.P. (1983). The liver in the IgA secretory immune system. Dogs, but not rats and rabbits, are suitable models for human studies. Hepatology **3:** 980–988.

Delacroix D.L., and Vaerman J.P. (1981). A solid phase, direct competition, radioimmunoassay for the quantitation of secretory IgA in human serum. J. Immunol. Methods **40:** 345–358.

Du Pasquier L., Weiss N., and Loor F. (1972). Direct evidence for immunoglobulins on the surface of thymus lymphocytes of amphibian larvae. Eur. J. Immunol. **2:** 366–370.

Fellah J.S. (1989). Les immunoglobulines de l'axolotl (Amphibien urodèle). Thèse de l'Université Pierre et Marie Curie, Paris.

Fellah J.S., and Charlemagne J. (1988). Characterization of an IgY-like low molecular weight immunoglobulin class in the Mexican axolotl. Molec. Immunol. **25:** 1377–1386.

Fellah J.S., Vaulot D., Tournefier A., and Charlemagne J. (1989). Ontogeny of immunoglobulin expression in the Mexican axolotl. Development **107:** 253–263.

Gendelman H.E., Moench T.R., Narayan O., and Griffin D.E. (1983). Selection of a fixative for identifying T cell subsets, B-cell and macrophages in paraffin-embedded mouse spleen. J. Immunol. Meth. **65:** 137–145.

Geneste C., Mangalo R., Iscaki S., and Pillot J. (1986). Human secretory component. IV. Antigenic regions involved in *in vitro* binding of dimeric IgA. Immunol. Lett. **13:** 121–126.

Hädge D., Fiebig H., Puskas E., and Ambrosius H. (1980). Evolution of low molecular weight immunoglobulins. I. Relationships of 7S immunoglobulins of various vertebrates to chicken IgY. Develop. Comp. Immunol. **4:** 510–513.

Hart S., Wrathmell A.B., Harris J.E., and Grayson T.H. (1988). Gut immunology in fish: a review. Develop. Comp. Immunol. **12:** 453–480.

Hirata I., Berribi G., Austin L.L., Keren D.F., and Dobbins W. (1986). Immunohistochemical characterization of intraepithelial and *lamina propria* lymphocytes in control ileum and colon and in inflammatory bowel disease. Dig. Dis. Sci. **31:** 593–606.

Hsu E., Flajnik M.F., and Du Pasquier L. (1985). A third immunoglobulin class in amphibians. J. Immunol. **135:** 1998–2004.

Iscaki S., Geneste C., and Pillot J. (1978). Human secretory component. I. Evidence for a new antigenic specificity. Immunochemistry **15:** 401–408.

Kühn L.C., and Kraehenbühl, J.P. (1979). Role of secretory component, a secreted glycoprotein, in the specific uptake of IgA dimer by epithelial cells. J. Biol. Chem. **254:** 11072–11081.

Kühn, L.C., and Kraehenbühl J.P. (1981). The membrane receptor for polymeric immunoglobulin is structurally related to secretory component. J. Biol. Chem. **256:** 12490–12495.

Lebacq-Verheyden A.M., Vaerman J.P., and Heremans, J.F. (1972). Immunohistologic distribution of the chicken immunoglobulins. J. Immunol. **109:** 652–654.

Lemaître-Coelho I., André C., and Vaerman J.P. (1977a). Murine secretory component. *Protides Biol. Fluids* **25:** 891–894.

Lemaître-Coelho I., Jackson G.D.F., and Vaerman J.P. (1977b). Rat bile as a convenient source of secretory IgA and free secretory component. Eur. J. Immunol. **7:** 588–590.

Mostov K.E., Friedlander M., and Blobel G. (1984). The receptor for transepithelial transport of IgA and IgM contains multiple immunoglobulin-like domains. Nature (London) **308:** 37–43.

Nagura H., Nakane P.K., and Brown W.R. (1979). Translocation of dimeric IgA through neoplastic colon cells in vitro. J. Immunol. **123:** 2359–2868.

Pabst R. (1987). The anatomical basis for the immune function of the gut. Anat. Embryol. **176:** 135–144.

Tomasi T.B., Tan E.M., Salomon A., and Prendergast R.A. (1965). Characteristics of an immune system common to certain external secretions. J. Exp. Med. **121:** 101–124.

Underdown B.J., and Schiff J.M. (1986). Immunoglobulin A: strategic defense initiative at the mucosal surface. Annu. Rev. Immunol. **4:** 389–417.

Vaerman J.P., Heremans J.F., Bazin H., and Beckers A. (1975a). Identification and some properties of rat secretory component. J. Immunol. **114:** 265–669.

Vaerman J.P., Naccache-Corbic M., and Heremans J.F. (1975b). Secretory component of the guinea pig. Immunology **29:** 933–940.

